# Progress in Surgery

**Published:** 1897-04-03

**Authors:** 


### Progress in Surgery.

### GENERAL SURGERY.

**Antiseptics.**—Wible's[1] experience with acetanilid (antefebrin) in different classes of wounds, burns and scalds, and amputations, has given results not only equal but superior in every respect to iodoform without its disadvantages. It is a cheap, non-irritating, non-poisonous, and inodorous form of surgical dressing, and has also some hemostatic properties; is strongly antiseptic and desiccant when applied to a fresh wound, burn, or scald in powder form, it combines with the wound secretion and forms a thin scab, under which repair rapidly progresses. The slight burning sensation caused by the powder soon ceases, and is then succeeded by its anæsthetic and analgesic effect. Crush-wounds of the extremities, where dust, dirt, and grease are ground into the lacerated tissues—and it is impossible to wash out all the foreign matter with antiseptic solutions —have been found by Wible to heal generally without suppuration when treated with acetanilid. A bacteriological inquiry into the relative value of various agents used in the disinfection of the hands has been carefully set out by Chas. Leedham-Green.[2] The conclusions he draws from his experiments are that the difficulty of sterilising the hands and skin has been very greatly under-estimated. It cannot be said that any of the methods at present in use may be relied upon for absolute sterilisation. The best results are likely to be gained by an extension of Fürbringer's process; and it must be allowed that, although the use of alcohol will not accomplish all, or nearly all, that Reinicke and others claim for it, it is nevertheless a valuable, if not the most valuable, agent we possess. Lauenstein[3] gives the results of prolonged and apparently carefully conducted bacteriological and clinical research upon the possibility of disinfecting the skin of an operation region. The author demonstrates the fact that the staphylococcus pyogenes albus is the microbe most frequently found in and on the skin, not only in Europeans, but practically in natives of all nations. After this the S. aureus follows in frequency of appearance. By present methods the surgeon cannot hope to disinfect the skin thoroughly. No matter how thorough and how long-continued the disinfection has been, infection may start from the skin, and under favouring circumstances reach the underlying parts and seriously complicate wound healing. Therefore, stitches which include the whole depth of a wound are unsafe, since infection may follow their course. The deeper parts of the wound should be closed by buried sutures and the skin stitches of wire or silkworm gut—not catgut—should include only the skin. The impossibility of skin disinfection also constitutes a strong reason for omitting drainage whenever this is possible, and for keeping the surface surrounding the wound dry. Popper[4] does not consider, from investigations he has made, that stitch suppuration at the Giessen clinic after the use of catgut is due to its infection with bacteria, but to some chemical matter. This suppuration is always of an innocent character, and has no tendency, like bacterial processes, to become diffuse. Phlegmonous inflammation, therefore, after operation, can no longer be attributed to the use of catgut, but to an accident, and due to bacteria. Hofmeister,[5] after referring to the different methods of catgut sterilisation, employed in Braun's clinic (Tübingen), recommends the following process, the value of which depends upon the capability of formalin of acting on lime substances, so that they lose their solubility in boiling water. Harden the raw catgut, which has been previously wrapped upon rolls, in 4 per cent. solution of formalin for twenty-four hours. Boil in water for ten minutes; harden again in the formalin solution, and preserve in alcohol, to which 5 per cent. of glycerine and 1 per cent. sublimate or other antiseptic in suitable quantity has been added. Bacteriological investigation showed the prepared catgut to be free from spores. A concise account of the surgical aspect of modern war is given in a recent number of the *Practitioner*.[6] It states that, for the prevention of wound infection, it is essential that in all efforts to render first aid in war two principles should predominate: (*a*) To cover the wounds as soon as possible with antiseptic material, the usual "first field dressing"; (*b*) to transfer the injured as rapidly as possible to places where they may receive surgical help. Extensive experience has confirmed the belief that a very great number even of the most severe gunshot wounds remain aseptic if not touched o examined by dirty fingers or instruments; although a thorough antiseptic treatment of gunshot or other wounds is impossible on the battle-field, yet the element of delay in treatment materially favours the prospects of infection. The general conservative treatment of injuries to the extremities in the field hospitals is now comparatively easy, as the equipment of the bearer companies and sections of the field hospitals provides an ample supply of antiseptics and surgical appliances.

**Fractures and Dislocations.**—In an able article on the treatment of fractures, Dr. G. G. Davis[7] does not support the practice of allowing patients to walk about on broken limbs before union has occurred. He agrees with Reclus in saying immobilise the fragments, but mobilise the muscles, tendons, and joints. Massage will improve the circulation, lessen the pain, tend to prevent adhesion of the tendons to their sheaths, promote the absorption of inflammatory material, and prevent contraction or atrophy of the muscles. Passive motion judiciously employed tends to prevent the limitation of the motion of joints by the formation of strong fibrous bands, and likewise to replace any displaced fragments of bone and keep the part in its normal shape. The importance of union and avoidance of deformity is so great that it is, as a rule, unwise to resort to massage or any marked movements until the fragments become to a certain degree fixed in the proper position, usually for the sixth to the tenth day, and in the lower extremity frequently much longer. Afterwards, the more frequent the movement and massage, if effected gently and without disturbance of the bones, the quicker the recovery. Daily massage will not be too often. Two cases of ununited fracture of the humerus treated according to the method already described in The Hospital by fixation with bone ferrules are published by N. Senn[8] from his surgical clinic, but the ultimate results are not established. The ferrules are absorbed in from six to eight weeks. In two skiagrams by J. Lynn Thomas[9] of Colles's fracture of the radius the often doubted concomitant fracture of the styloid process is well depicted. He found this condition of the ulnar styloid process in four out of five cases skiagraphed. The view that fracture of this process and rupture of the triangular fibro-cartilage of the wrist are frequent complications is also reintroduced by R. C. Lucas,[10] who quotes from his paper in the Guy's Hospital Reports for 1884, that "one cannot fail to be struck with a certain analogy between Colles's fracture of the upper extremity and Potts' fracture of the lower." Forward displacement of the lower fragment in Colles's fracture, although believed to be of the rarest occurrence, is proved by J. B. Roberts[11] to be not infrequent. In his interesting article he describes the causes and mechanism, as well as the symptoms and diagnosis of this injury, at the same time depicting many of the museum specimens in this country as well as in America. The treatment consists in immediate and complete reduction of the fracture, followed by a retentive splint, which will not interfere with the free use of the fingers. The deformity must be overcome, even if great force is necessary to loosen the impaction, by the surgeon grasping the metacarpus of the patient with one hand, the lower part of the forearm with the other. This should be done with the patient's hand in the supine position. The thumb of the surgeon's hand which holds the metacarpus should be placed on the palmar surface of the carpal fragment of the radius, as it lies just behind the thenar eminence. Extension and counter-extension are made for a moment; the hand suddenly bent backward in strong dorsal flexion, and the lower fragment at the same instant pushed backwards into place by the surgeon's thumb. Reduction is painful, but is so quickly done that, as a rule, anæsthesia is unnecessary. A coarse, grating sensation is experienced. Occasionally a repetition of the manipulation may be needed to obtain perfect restoration of the bony contour. In old dislocations of the elbow W. J. Welsh[12] recommends cutting into the joint, severing the tendon of the triceps, removing the olecranon, dissecting them both out, and then, by proper and careful manipulation, bringing the radius and ulna forwards into their proper places, and retaining them there with the view of establishing ankylosis. Intractable ankylosis need not be feared, as time and use will largely restore the functions of the limb and joint, including rotation of the forearm. Kofend[13] records an unique case of persistent traumatic dislocation of both hips. The patient was admitted for malignant disease of the lower jaw, but the special interest referred to the condition of the lower limbs. Thirteen years before, at 42 years of age, the hip joints were dislocated, and treated unsuccessfully by a bone-setter. On examination, the right thigh was flexed, adducted, and rotated in, the foot pointing forwards, and much flattening in front of the acetabulum, but the head and trochanter could be easily felt behind. The head was four inches above Nélaton's line, and the head lay in a capsule somewhat above and in front of the great sciatic foramen. The acetabulum could not be felt. The left thigh was almost at right angles to the body, the head being beneath the obturator foramen, so that its movements could readily be felt per rectum. The patient stood mainly on the right foot, the pelvis tilted to the left and forwards, and the left limb in the position of genu varum. There was compensatory lordosis and scoliosis. The gait was oscillatory, and walking only possible with the aid of a stick in the right hand. Operation for the relief of the deformity was refused. Two cases of the somewhat rare lateral dislocation outwards of the knee-joint are figured and recorded by F. Page;[14] these are of interest inasmuch as no drawings of this injury are given in recent text-books on surgery. D. Graham[15] relates four cases of displaced semilunar cartilage treated by massage, movement, and manipulation. He concludes by stating that in attempting to replace the cartilage, the knee should be flexed, then extended suddenly, the leg rotated inwards if it be the internal, outwards if it be the external, while pressure is exerted over the injured region. After a meniscus has been replaced it is secured by a pad and figure of eight bandage—instead of plaster and splints. Carefully applied massage, resistive movements, home exercises, and electricity so strengthen the front muscles of the thigh, the fascia, ligaments and attachments of the knee-joint that they will safely hold a previously dislocated semilunar cartilage without artificial support. These cases do not include those requiring surgical operation. The following case, recorded by W. Thorburn,[16] is of interest as indicating the value of the X rays in diagnosis and because the lesion discovered is probably unique. A labourer, aged 61, in good general health, had a weight fall on his left foot, immediately behind the first metatarso-phalangeal joint. For three weeks he continued at work with continuous pain, but thereafter totally incapacitated by increasing pain on standing and walking. By an incision the external sesamoid bone attached to the tendons of the flexors of the great toe was exposed, found to be displaced outwards from its articulation with the metatarsal bone (? and excised). The wound healed by primary union, and two months later the patient was again at work.

[1] Therapeutic Gazette, Oct. 15, 1896, p. 692. [2] Brit. Med. Journ., Oct. 17, 1896, p. 1,109. [3] Therapeutic Gazette, Oct. 15, 1896, p. 669; and Archiv. für Klin. Chir., bd. 53, heft 1. [4] Epit. Brit. Med. Journ., Jan. 9, 1897; and Deut. Med. Woch. No. 48, 1896. [5] Amer. Jour. of the Med. Sciences, Sept., 1896; and Centralblatt für Chir, 1896. [6] Practitioner, Sept., 1896, p. 276. [7] Annals of Surgery, Dec., 1896, p. 691. [8] Chicago Clinical Review, Sept., 1896, p. 676. [9] Brit. Med. Jour., Jan. 2, 1897, p. 12. [10] Brit. Med. Jour., Jan. 9, 1897, p. 113. [11] Amer Jour. of the Med. Sciences, Jan. 1897, p. 10. [12] New York Med. Record, Sept. 19, 1896, p. 425. [13] Epit. B. M. Jour., Nov. 14, 1896; Wien. Klin. Woch., Aug. 20, 1896. [14] Lancet, Oct. 3, 1896, p. 938. [15] Amer. Journ. of the Med. Sciences, Nov. 1896, p. 561. [16] Medical Chronicle, Nov. 1896, p. 91.

